# AMP-activated protein kinase mediates VEGF-stimulated endothelial NO production

**DOI:** 10.1016/j.bbrc.2007.01.110

**Published:** 2007-03-23

**Authors:** James A. Reihill, Marie-Ann Ewart, D. Grahame Hardie, Ian P. Salt

**Affiliations:** a Division of Biochemistry and Molecular Biology, Institute of Biomedical and Life Sciences, University of Glasgow, Glasgow G12 8QQ, UK; b Division of Molecular Physiology, College of Life Sciences, Sir James Black Centre, University of Dundee, Dundee DD1 5EH, UK

**Keywords:** Endothelium, Vascular endothelial growth factor, AMP-activated protein kinase, Nitric oxide

## Abstract

Vascular endothelial growth factor (VEGF) is an important regulator of endothelial cell function. VEGF stimulates NO production, proposed to be a result of phosphorylation and activation of endothelial NO synthase (eNOS) at Ser1177. Phosphorylation of eNOS at this site also occurs after activation of AMP-activated protein kinase (AMPK) in cultured endothelial cells. We therefore determined whether AMPK mediates VEGF-stimulated NO synthesis in endothelial cells. VEGF caused a rapid, dose-dependent stimulation of AMPK activity, with a concomitant increase in phosphorylation of eNOS at Ser1177. Infection of endothelial cells with an adenovirus expressing a dominant negative mutant AMPK partially inhibited both VEGF-stimulated eNOS Ser1177 phosphorylation and NO production. VEGF-stimulated AMPK activity was completely inhibited by the Ca^2+^/calmodulin-dependent protein kinase kinase inhibitor, STO-609. Stimulation of AMPK via Ca^2+^/calmodulin-dependent protein kinase kinase represents a novel signalling mechanism utilised by VEGF in endothelial cells that contributes to eNOS phosphorylation and NO production.

VEGF is a key regulator of angiogenesis, stimulating differentiation, survival, migration, proliferation, and vascular permeability of endothelial cells [1,2]. VEGF binds to VEGF receptor tyrosine kinases, which have been demonstrated to stimulate a diverse array of signalling pathways, including phospholipase C (PLC)-γ, phosphatidylinositol 3′-kinase (PI3K), and Src [1,2].

VEGF rapidly stimulates endothelial NO synthesis [3–5], proposed to be the result of PI3K-mediated activation of protein kinase B (PKB, also known as Akt), which directly phosphorylates and activates eNOS at Ser1177 [4,6]. However, VEGF-stimulated NO production has been reported to be only partially inhibited by wortmannin, suggesting that VEGF-stimulated NO production is mediated by both PI3K/PKB and a PI3K-independent kinase [3,5,7].

In addition to PKB, AMPK has been shown to phosphorylate and activate eNOS at Ser1177 in cultured endothelial cells [8,9]. AMPK is activated by phosphorylation at Thr172 by at least two recently characterised upstream kinases, LKB1 and Ca^2+^/calmodulin-dependent protein kinase kinase (CaMKK), especially the β isoform [10–13]. It has been proposed that the phosphorylation of AMPK by LKB1 is triggered by metabolic stresses that increase the intracellular AMP:ATP ratio [10,11], while phosphorylation and activation of AMPK by CaMKK is Ca^2+^-dependent and independent of changes in cellular AMP [11–13]. A key role of AMPK in the mediation of eNOS phosphorylation has been demonstrated in response to hypoxia, metformin, adiponectin, and shear stress [9,14–17]. In addition, AMPK has been proposed to mediate angiogenesis stimulated by adiponectin and hypoxia [15,18]. AMPK, therefore, represents a candidate PI3K-independent kinase that underlies VEGF-stimulated eNOS phosphorylation. In this study, we have investigated whether VEGF-stimulated NO production is mediated, in part, by AMPK in cultured human aortic endothelial cells (HAECs).

## Methods

*Materials*. HAECs and endothelial cell culture media were obtained from TCS Cellworks (Botolph Claydon, Bucks, UK). VEGF_165_ and U73122 were supplied by Sigma (Poole, Dorset, UK). STO-609 was from Tocris (Ellisville, MO, USA). PP1 was a generous gift from Prof. G. Milligan, University of Glasgow, UK Rabbit anti-acetyl CoA carboxylase (ACC) and anti-phospho-ACC Ser79 (rat ACC1 sequence) antibodies were supplied by Upstate (Lake Placid NY, USA). Goat anti-CaMKKα and mouse anti-CaMKKβ antibodies were supplied by Santa Cruz Biotechnology (Heidelberg, Germany). Rabbit anti-phospho-AMPK Thr172 antibodies were obtained from Cell Signaling Technology (Beverly MA, USA). SAMS peptide (HMRSAMSGLHLVKRR) was synthesised by Pepceuticals Ltd. (Nottingham, UK). All other reagents were from sources described previously [9].

*Cell culture*. HAECs were grown in large vessel endothelial cell medium at 37 °C in 5% CO_2_ and used for experiments between passages 3 and 6.

*Preparation of cell lysates and AMPK assay*. Cells were preincubated for 2 h at 37 °C in 5 ml Krebs Ringer Hepes (KRH) buffer (119 mM NaCl, 20 mM Hepes, pH 7.4, 5 mM NaHCO_3_, 4.7 mM KCl, 1.3 mM CaCl_2_, 1.2 mM MgSO_4_, 1 mM KH_2_PO_4_, 0.1 mM l-arginine, 5 mM glucose). After addition of test substances for various durations, lysates were prepared, AMPK was immunoprecipitated and assayed using the SAMS substrate peptide as described previously [9].

*Adenoviruses and infection of HAECs*. Control (Ad.Null) and recombinant adenovirus expressing dominant negative AMPK (Ad.α1DN) were generous gifts from Dr. F. Foufelle, Centre Biomédical des Cordeliers, Paris. Viruses were propagated, purified, and stored as described previously [9]. HAECs were infected with 25 Pfu/cell adenovirus and cultured for 24 h prior to experimentation. Within 24 h of infection with a GFP-expressing virus, the majority (>95%) of HAECs expressed GFP as previously described [9].

*Assay of NO production in endothelial cells*. Cells cultured in 6-well plates were preincubated for 2 h at 37 °C in 0.5 ml/well KRH buffer at 37 °C. The medium was removed and replaced with 0.4 ml fresh KRH buffer in the presence or absence of VEGF (10 ng/ml), wortmannin (100 nM) and/or l-NAME (1 mM) for 15 min. The medium was removed, NO-specific chemiluminescence was analysed and l-NAME-sensitive NO production calculated as described previously [9].

*Immunoprecipitation of CaMKKβ*. HAEC lysates (100 μg) were added to 0.5 μg mouse anti-CaMKKβ antibody and mixed overnight at 4 °C. Protein G–Sepharose (20 μl of 25% slurry) was added, the volume adjusted to 300 μl with lysis buffer and mixed for 4 h at 4 °C. The mixture was centrifuged (14,000*g*, 30 s, 4 °C) and the pellet was washed three times in lysis buffer.

*Statistics*. Unless stated otherwise, results are expressed as means ± SD. Statistically significant differences were determined using a Student’s *t*-test, using *p* < 0.05 as significant using Statview software.

## Results and discussion

**Fig. 1:**
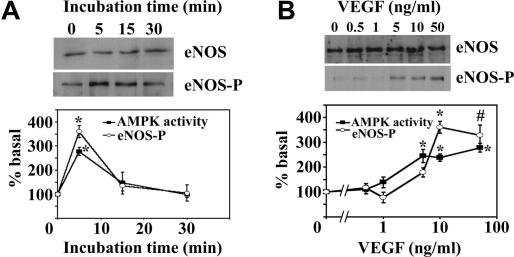
Effect of VEGF on AMPK activity and eNOS Ser1177 phosphorylation. (A) HAECs were incubated with 10 ng/ml VEGF for various times and lysates prepared. (B) HAECs were incubated with the indicated concentrations of VEGF for 5 min and lysates prepared. Total AMPK was immunoprecipitated from endothelial cell lysates and assayed for AMPK activity. In parallel experiments, lysates were subjected to SDS–PAGE, transferred to nitrocellulose and probed with the antibodies indicated. The intensity of the resultant bands was quantified using NIH Image software. Representative immunoblots are shown, repeated with similar results on three different samples of lysates. The results are expressed as the means ± SD % basal AMPK activity (■) or % basal eNOS phosphorylation (○) for three independent experiments. *p < 0.01 relative to value in absence of VEGF. ^#^*p* < 0.05 relative to value in absence of VEGF.

To investigate whether AMPK mediates VEGF-stimulated NO synthesis, we first determined the effects of physiological concentrations of VEGF on AMPK activity and eNOS Ser1177 phosphorylation in HAECs. VEGF (10 ng/ml) elicited a transient, concomitant activation of AMPK and eNOS phosphorylation at Ser1177, which reached a maximum 2.8- and 3.6-fold stimulation, respectively, after 5 min. Both AMPK activity and eNOS phosphorylation rapidly returned to basal levels (Fig. 1A). In addition, activation of AMPK and phosphorylation of eNOS at Ser1177 by VEGF shared a similar dose-dependence, such that AMPK activity and eNOS Ser1177 phosphorylation were stimulated maximally (2.5- and 3.6-fold, respectively) by 10 ng/ml VEGF, a concentration at which all further experiments were performed (Fig. 1B). Previous studies have reported that stimulation of human umbilical vein endothelial cells (HUVECs) with VEGF for 6 h or bovine aortic endothelial cells (BAECs) with VEGF for 10 min was without effect on phosphorylation of AMPK at Thr172 [15,19]. These studies are in agreement with the present study in which VEGF-stimulated AMPK activity peaked after 5 min incubation and returned to basal values within 15 min.

**Fig. 2:**
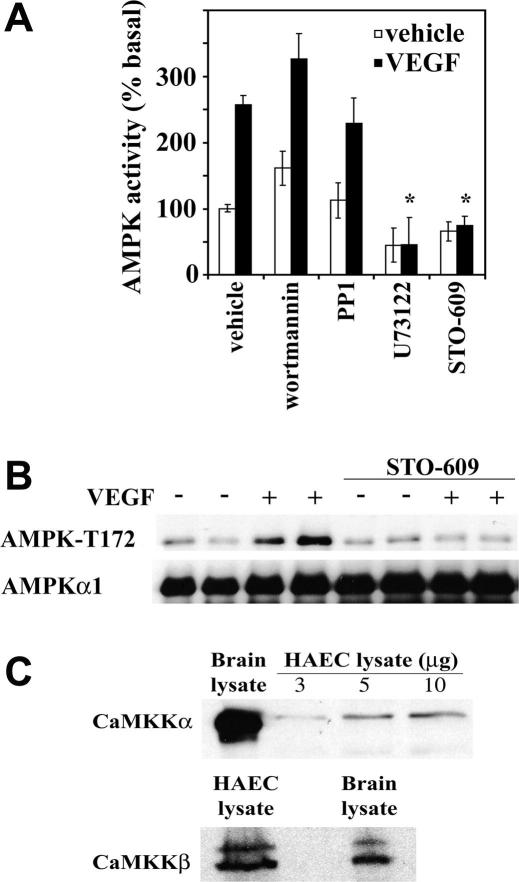
Effect of inhibitors of PLC and CaMKK on VEGF-stimulated AMPK activity. HAECs were incubated in the presence or absence of 10 ng/ml VEGF for 5 min after preincubation with 100 nM wortmannin, 1 μM PP1, 10 μM U73122 or 25 μM STO-609 for 45 min and lysates prepared. (A) Total AMPK was immunoprecipitated from HAEC lysates and assayed for AMPK activity. The results are expressed as the means ± SD % basal AMPK activity for four independent experiments. *p < 0.01 relative to value in absence of inhibitor. (B) AMPK immunoprecipitates were resolved by SDS–PAGE, transferred to nitrocellulose and probed with the antibodies indicated. Representative immunoblots are shown, repeated with similar results on four different samples of lysates. (C) HAEC lysates were resolved by SDS–PAGE, transferred to nitrocellulose and probed with anti-CaMKKα antibodies. CaMKKβ was immunoprecipitated from HAEC lysate, subjected to Western blotting and probed with anti-CaMKKβ antibodies. A lysate prepared from whole rat brain was used as a positive control. Representative immunoblots are shown.

PI3K, PLC, and c-Src are known effectors of VEGF signalling in endothelial cells and previous studies have suggested that AMPK stimulation is downstream of PI3K and c-Src activation in peroxynitrite- and metformin-treated BAECs [16,19]. In the current study, inhibition of either PI3K or c-Src had no effect on VEGF-stimulated AMPK activity in HAECs, suggesting that neither PI3K nor c-Src mediate VEGF-stimulated AMPK activation (Fig. 2A). Preincubation with U73122 completely inhibited VEGF-stimulated AMPK activity (Fig. 2), suggesting that VEGF stimulates AMPK in a PLC-dependent manner. Similarly, histamine has previously been reported to activate AMPK in HUVECs in a PI3K-independent, PLC-dependent manner [20].

It is now apparent that CaMKKβ can act as an alternate upstream kinase to LKB1 that activates AMPK in a Ca^2+^-dependent and AMP-independent manner [11–13]. A recent report indicates that CaMKK mediates thrombin-stimulated AMPK activation in HUVECs, but that AMPK does not underlie thrombin-stimulated eNOS Ser1177 phosphorylation [21]. As VEGF is known to stimulate PLCγ-mediated increases in intracellular Ca^2+^ [3,22] and VEGF-stimulated AMPK activity is inhibited by the PLC inhibitor U73122 (Fig. 2A), we examined whether VEGF-stimulated AMPK activity was mediated by CaMKK activation by using the CaMKK inhibitor, STO-609. Preincubation of HAECs with STO-609 reduced VEGF-stimulated AMPK activity to basal levels (Fig. 2A), without altering AICAR-stimulated AMPK activity, which is mediated by LKB1 (data not shown). VEGF-stimulated AMPK Thr172 phosphorylation was similarly reduced to basal levels after preincubation with STO-609 (Fig. 2B). STO-609 inhibits both CaMKKα and CaMKKβ, yet the expression of these isoforms in endothelial cells has not previously been determined. Using isoform-specific anti-CaMKK antibodies, we demonstrated expression of both CaMKKα and CaMKKβ in HAECs (Fig. 2C). These data indicate that VEGF stimulates CaMKK via PLC-mediated Ca^2+^ mobilisation, and that CaMKK, rather than LKB1 is the upstream kinase responsible for AMPK activation in response to VEGF in endothelial cells.

**Fig. 3:**
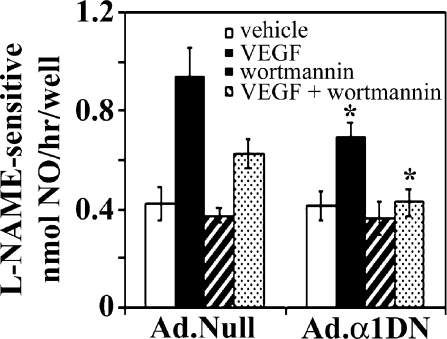
The effects of infection with Ad.α1DN on VEGF-stimulated NO production. HAECs were infected with 25 Pfu/cell of Ad.Null or Ad.α1DN viruses 24 h prior to experimentation. Cells were incubated in KRH buffer in the presence or absence of 10 ng/ml VEGF_165_ and/or 100 nM wortmannin. After 15 min, medium was removed and assayed for l-NAME-sensitive ${\text{NO}}_{2}^{-}$ content. The data shown represent the means ± SD NO synthesis from nine independent experiments. *p < 0.05 relative to value in Ad.Null-infected cells.

**Fig. 4:**
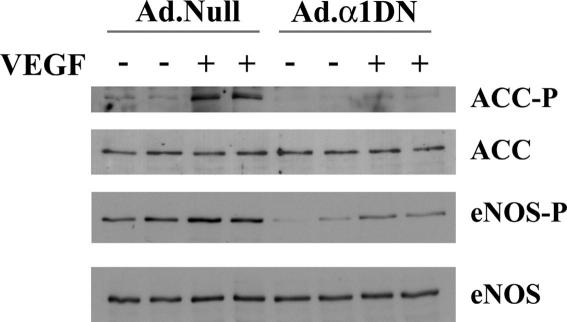
The effects of infection with Ad.α1DN on VEGF-stimulated ACC and eNOS phosphorylation. HAECs were infected with 25 Pfu/cell of Ad.α1DN or Ad.Null 24 h prior to experimentation. Subsequently, HAEC lysates were prepared from cells incubated in the presence or absence of 10 ng/ml VEGF, resolved by SDS–PAGE, transferred to nitrocellulose and probed with the antibodies indicated. Specific band intensities were quantified using NIH Image software. Representative immunoblots are shown, repeated with similar results on four different samples of lysates.

To determine the functional effects of VEGF-stimulated AMPK activity, we investigated the role of AMPK in VEGF-stimulated eNOS phosphorylation and NO production. Previous studies have proposed that VEGF-stimulated eNOS phosphorylation at Ser1177 is mediated by PKB [5,6], yet complete inhibition of PKB with the PI3K inhibitor, wortmannin only partially inhibited VEGF-stimulated NO production [3,5,7]. In agreement with this, preincubation of HAECs with the PI3K inhibitor wortmannin incompletely but significantly reduced VEGF-stimulated NO production by ∼65% (Fig. 3) at a concentration that completely inhibited phosphorylation of PKB at Ser473 (data not shown). These data suggest that both PI3K/PKB and a PI3K-independent kinase mediate VEGF-stimulated NO production. Infection of HAECs with Ad.α1DN caused a significant (∼40%) reduction in VEGF-stimulated NO production (Fig. 3). In the presence of wortmannin, infection with Ad.α1DN significantly reduced VEGF-stimulated NO production to basal levels. Quantification of eNOS phosphorylation status in Ad.α1DN-infected cells revealed VEGF-stimulated phosphorylation at Ser1177 was significantly reduced by approximately 70% (Fig. 4).

ACC is phosphorylated by AMPK at Ser80 in ACC1 and Ser220 in ACC2 (human sequence). Using an antibody that recognises both phosphorylated species, we demonstrated that VEGF stimulates phosphorylation of ACC, an effect completely inhibited in HAECs infected with Ad.α1DN (Fig. 4). These data indicate that infection with Ad.α1DN completely inhibits VEGF-stimulated AMPK activity. We were unable to distinguish whether the band represented ACC1, ACC2 or both. Inhibition of ACC1/ACC2 by phosphorylation at Ser80/Ser221 has been demonstrated to inhibit fatty acid synthesis in adipose tissue and liver whilst stimulating fatty acid oxidation in heart and skeletal muscle [11]. Activation of AMPK with AICAR has been shown to stimulate fatty acid oxidation in HUVECs [23], so it remains possible that VEGF transiently stimulates fatty acid oxidation due to AMPK-mediated phosphorylation of ACC.

Inhibition of VEGF-stimulated eNOS Ser1177 phosphorylation has previously been reported in HUVECs infected with adenoviruses expressing either dominant negative PKB or dominant negative AMPK under conditions of hypoxia, while under normoxic conditions, dominant negative AMPK was without any effect [15]. In contrast, we have demonstrated that AMPK contributes to VEGF-stimulated eNOS phosphorylation and NO production under normoxic conditions (Figs. 3 and 4). Given the effects of wortmannin and Ad.α1DN, we propose that VEGF stimulates both PKB and AMPK-mediated phosphorylation of eNOS at Ser1177 under normoxic conditions. Similarly, both PKB and AMPK-mediated phosphorylation of eNOS at Ser1177 has previously been suggested to occur in response to adiponectin in HUVECs [18]. As incubation of HAECs with wortmannin does not alter AMPK activity, AMPK does not act downstream of PI3K in the response to VEGF in HAECs (Fig. 2). It remains possible that AMPK could act upstream of PKB in VEGF-stimulated eNOS phosphorylation, but this seems unlikely because we have previously demonstrated that infection with Ad.α1DN does not alter PKB phosphorylation in HAECs [9].

In conclusion, we have demonstrated for the first time that VEGF stimulates the transient activation of AMPK in cultured endothelial cells in a PLC- and CaMKK-dependent manner. AMPK, therefore, represents a novel component of VEGF signalling. In addition, we propose that AMPK represents the PI3K-independent kinase that contributes, along with activated PKB to VEGF-stimulated eNOS Ser1177 phosphorylation and subsequent NO production.
